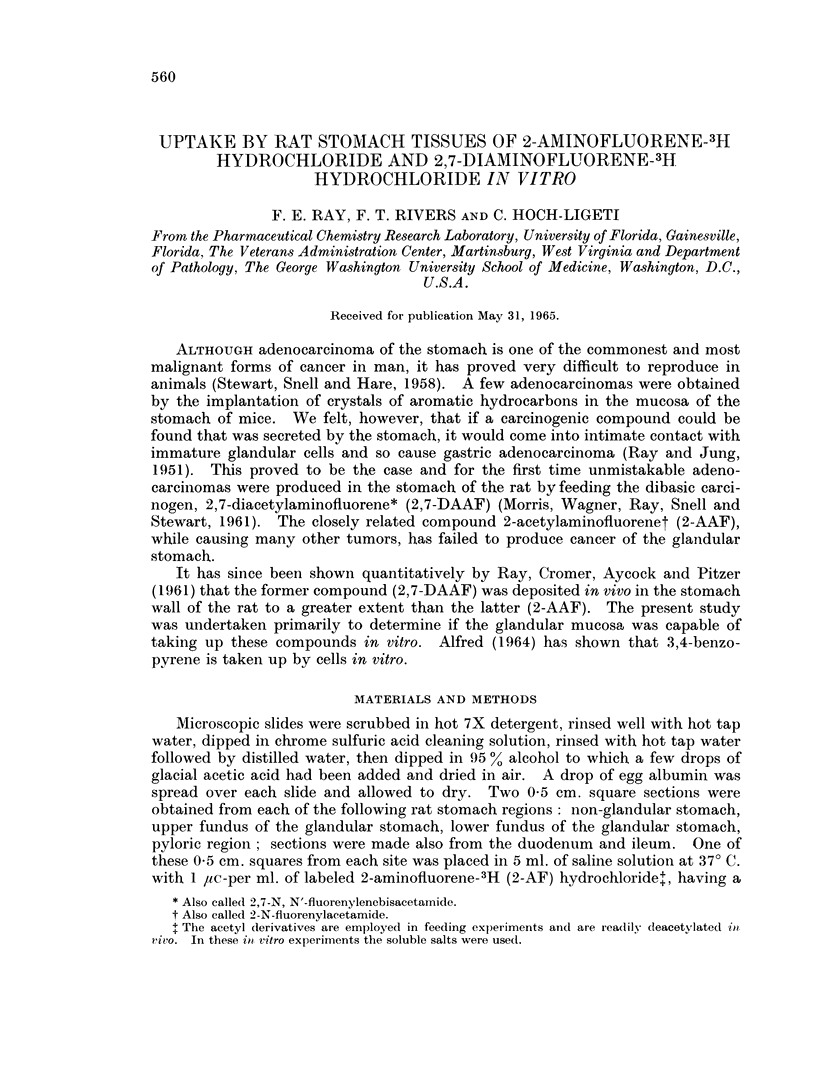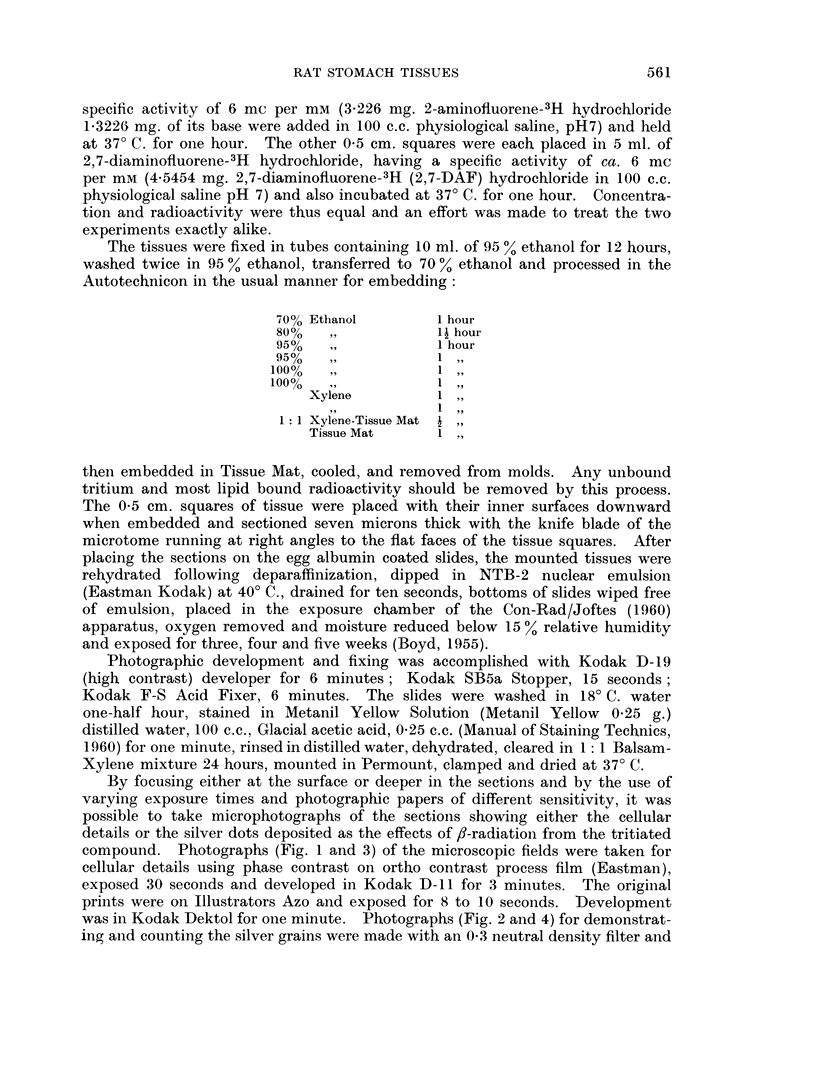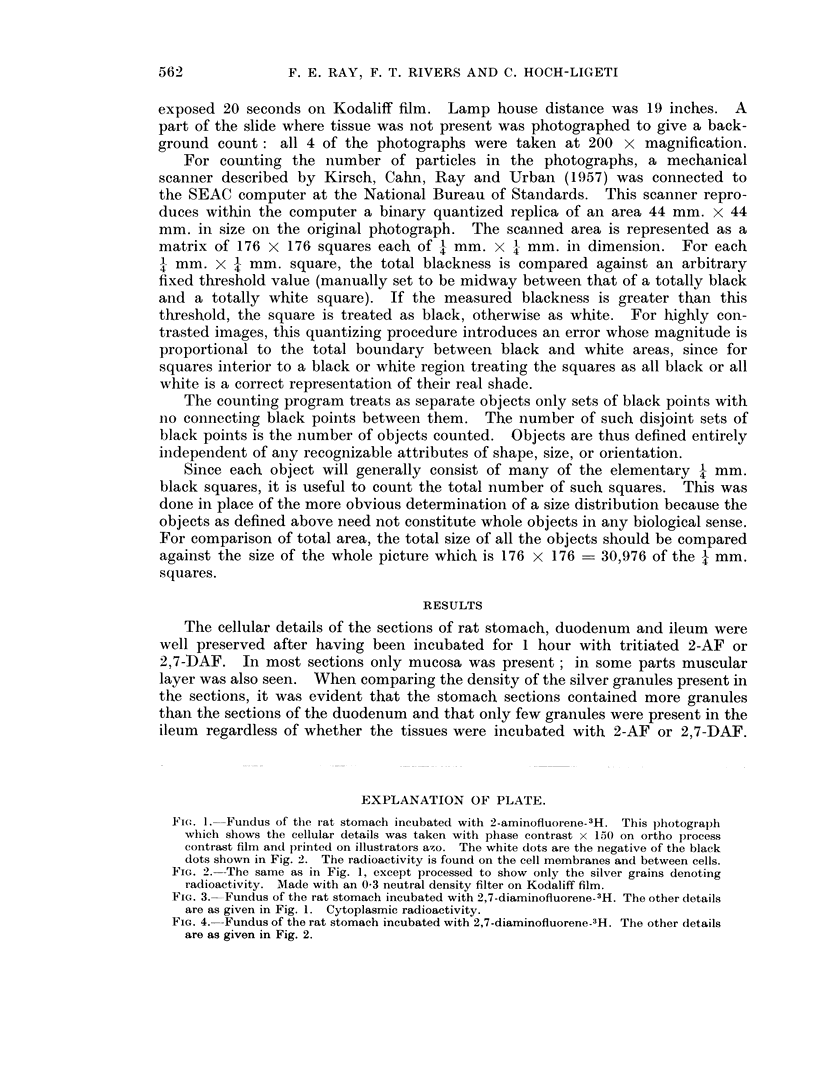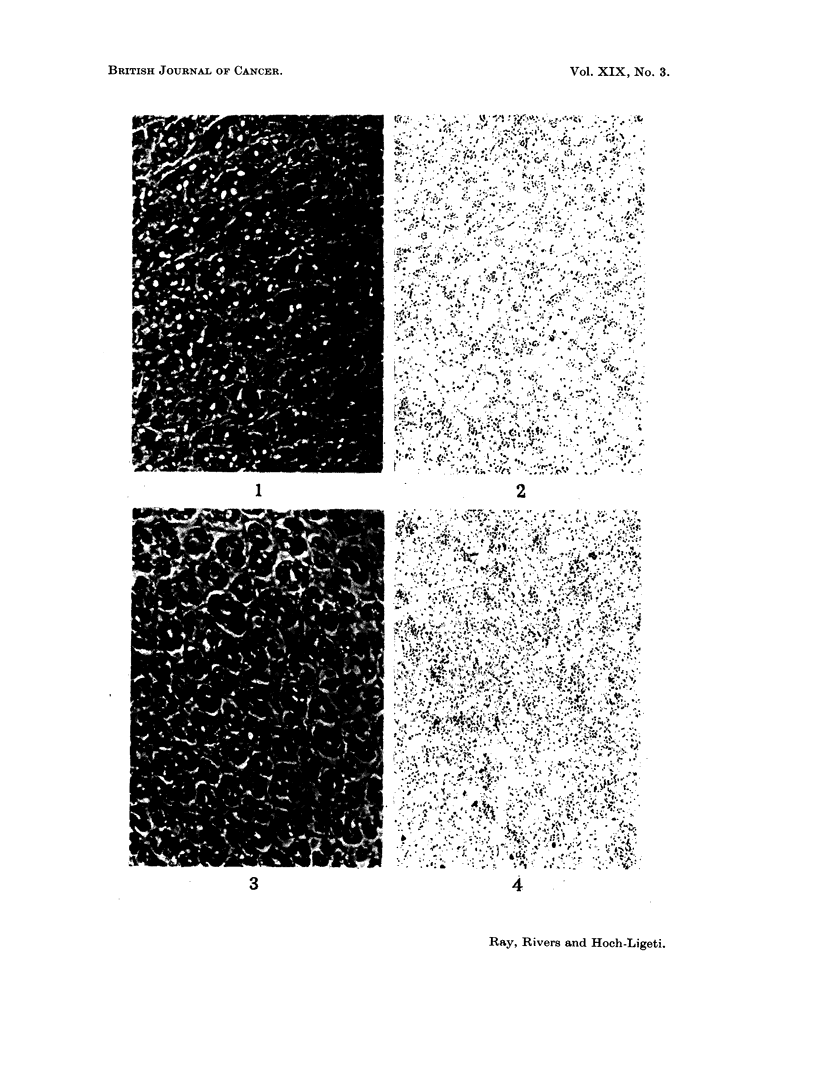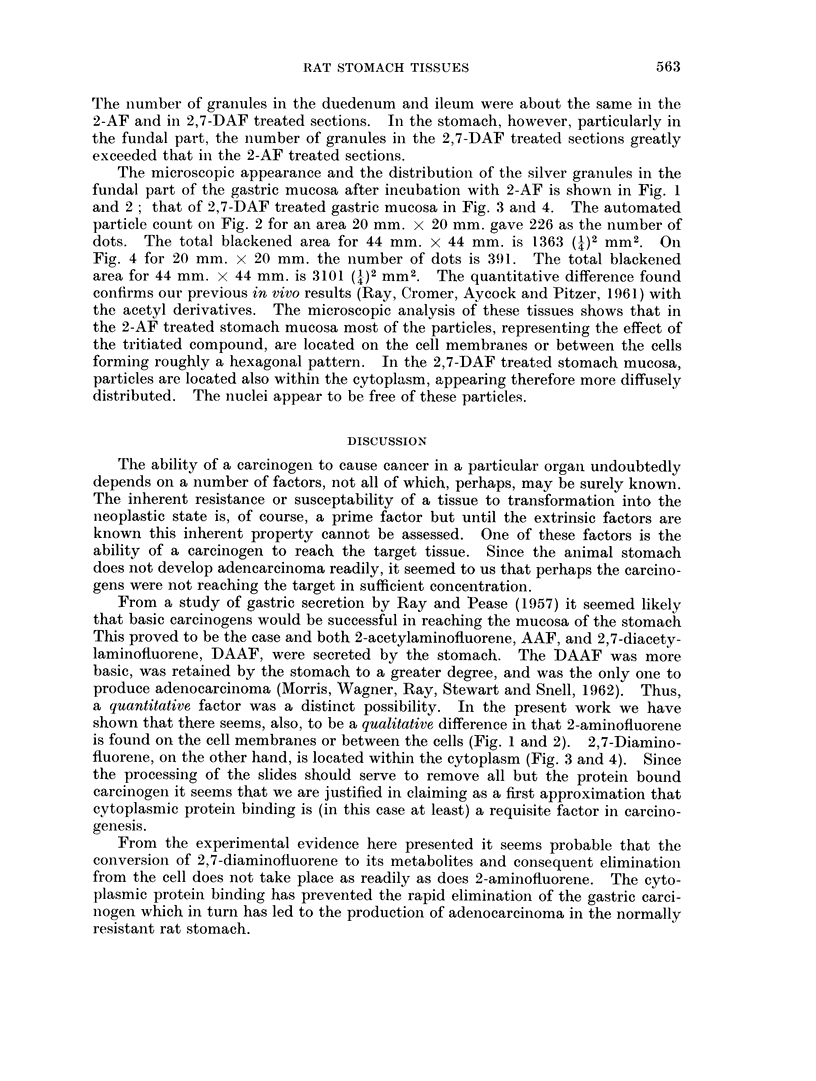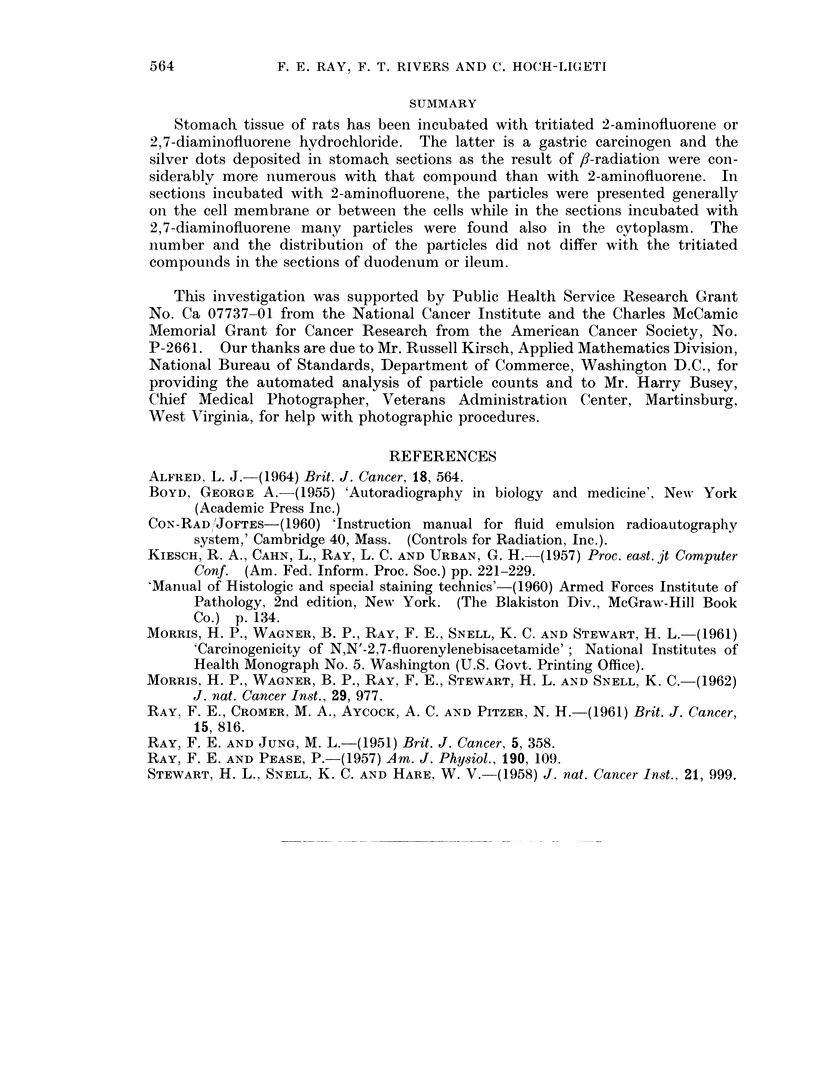# Uptake by rat stomach tissues of 2-aminofluorene-3H hydrochloride and 2,7-diaminofluorene-3H hydrochloride in vitro.

**DOI:** 10.1038/bjc.1965.69

**Published:** 1965-09

**Authors:** F. E. Ray, F. T. Rivers, C. Hoch-Ligeti

## Abstract

**Images:**


### UPTAKE BY RAT STOMACH TISSUES OF 2-AMINOFLUORENE-$^3$H HYDROCHLORIDE AND 2,7-DIAMINOFLUORENE-$^3$H HYDROCHLORIDE *IN VITRO*

F. E. RAY, F. T. RIVERS AND C. HOCH-LIGETI

*From the Pharmaceutical Chemistry Research Laboratory, University of Florida, Gainesville, Florida, The Veterans Administration Center, Martinsburg, West Virginia and Department of Pathology, The George Washington University School of Medicine, Washington, D.C., U.S.A.*

Received for publication May 31, 1965.

ALTHOUGH adenocarcinoma of the stomach is one of the commonest and most malignant forms of cancer in man, it has proved very difficult to reproduce in animals (Stewart, Snell and Hare, 1958). A few adenocarcinomas were obtained by the implantation of crystals of aromatic hydrocarbons in the mucosa of the stomach of mice. We felt, however, that if a carcinogenic compound could be found that was secreted by the stomach, it would come into intimate contact with immature glandular cells and so cause gastric adenocarcinoma (Ray and Jung, 1951). This proved to be the case and for the first time unmistakable adenocarcinomas were produced in the stomach of the rat by feeding the dibasic carcinogen, 2,7-diacetylaminofluorene* (2,7-DAAF) (Morris, Wagner, Ray, Snell and Stewart, 1961). The closely related compound 2-acetylaminofluorene† (2-AAF), while causing many other tumors, has failed to produce cancer of the glandular stomach.

It has since been shown quantitatively by Ray, Cromer, Aycock and Pitzer (1961) that the former compound (2,7-DAAF) was deposited *in vivo* in the stomach wall of the rat to a greater extent than the latter (2-AAF). The present study was undertaken primarily to determine if the glandular mucosa was capable of taking up these compounds *in vitro*. Alfred (1964) has shown that 3,4-benzopyrene is taken up by cells *in vitro*.

### MATERIALS AND METHODS

Microscopic slides were scrubbed in hot 7X detergent, rinsed well with hot tap water, dipped in chrome sulfuric acid cleaning solution, rinsed with hot tap water followed by distilled water, then dipped in 95% alcohol to which a few drops of glacial acetic acid had been added and dried in air. A drop of egg albumin was spread over each slide and allowed to dry. Two 0·5 cm. square sections were obtained from each of the following rat stomach regions : non-glandular stomach, upper fundus of the glandular stomach, lower fundus of the glandular stomach, pyloric region ; sections were made also from the duodenum and ileum. One of these 0·5 cm. squares from each site was placed in 5 ml. of saline solution at 37° C. with 1 $\mu$c-per ml. of labeled 2-aminofluorene-$^3$H (2-AF) hydrochloride‡, having a

* Also called 2,7-N, N′-fluorenylenebisacetamide.

† Also called 2-N-fluorenylacetamide.

‡ The acetyl derivatives are employed in feeding experiments and are readily deacetylated *in vivo*. In these *in vitro* experiments the soluble salts were used.

specific activity of 6 mc per mM (3·226 mg. 2-aminofluorene-$^3$H hydrochloride 1·3226 mg. of its base were added in 100 c.c. physiological saline, pH7) and held at 37° C. for one hour. The other 0·5 cm. squares were each placed in 5 ml. of 2,7-diaminofluorene-$^3$H hydrochloride, having a specific activity of *ca.* 6 mc per mM (4·5454 mg. 2,7-diaminofluorene-$^3$H (2,7-DAF) hydrochloride in 100 c.c. physiological saline pH 7) and also incubated at 37° C. for one hour. Concentration and radioactivity were thus equal and an effort was made to treat the two experiments exactly alike.

The tissues were fixed in tubes containing 10 ml. of 95% ethanol for 12 hours, washed twice in 95% ethanol, transferred to 70% ethanol and processed in the Autotechnicon in the usual manner for embedding :

| | | |
|---|---|---|
| 70% | Ethanol | 1 hour |
| 80% | ,, | 1½ hour |
| 95% | ,, | 1 hour |
| 95% | ,, | 1 ,, |
| 100% | ,, | 1 ,, |
| 100% | ,, | 1 ,, |
| | Xylene | 1 ,, |
| | ,, | 1 ,, |
| 1 : 1 | Xylene-Tissue Mat | ½ ,, |
| | Tissue Mat | 1 ,, |

then embedded in Tissue Mat, cooled, and removed from molds. Any unbound tritium and most lipid bound radioactivity should be removed by this process. The 0·5 cm. squares of tissue were placed with their inner surfaces downward when embedded and sectioned seven microns thick with the knife blade of the microtome running at right angles to the flat faces of the tissue squares. After placing the sections on the egg albumin coated slides, the mounted tissues were rehydrated following deparaffinization, dipped in NTB-2 nuclear emulsion (Eastman Kodak) at 40° C., drained for ten seconds, bottoms of slides wiped free of emulsion, placed in the exposure chamber of the Con-Rad/Joftes (1960) apparatus, oxygen removed and moisture reduced below 15% relative humidity and exposed for three, four and five weeks (Boyd, 1955).

Photographic development and fixing was accomplished with Kodak D-19 (high contrast) developer for 6 minutes ; Kodak SB5a Stopper, 15 seconds ; Kodak F-S Acid Fixer, 6 minutes. The slides were washed in 18° C. water one-half hour, stained in Metanil Yellow Solution (Metanil Yellow 0·25 g.) distilled water, 100 c.c., Glacial acetic acid, 0·25 c.c. (Manual of Staining Technics, 1960) for one minute, rinsed in distilled water, dehydrated, cleared in 1 : 1 Balsam-Xylene mixture 24 hours, mounted in Permount, clamped and dried at 37° C.

By focusing either at the surface or deeper in the sections and by the use of varying exposure times and photographic papers of different sensitivity, it was possible to take microphotographs of the sections showing either the cellular details or the silver dots deposited as the effects of $\beta$-radiation from the tritiated compound. Photographs (Fig. 1 and 3) of the microscopic fields were taken for cellular details using phase contrast on ortho contrast process film (Eastman), exposed 30 seconds and developed in Kodak D-11 for 3 minutes. The original prints were on Illustrators Azo and exposed for 8 to 10 seconds. Development was in Kodak Dektol for one minute. Photographs (Fig. 2 and 4) for demonstrating and counting the silver grains were made with an 0·3 neutral density filter and exposed 20 seconds on Kodaliff film. Lamp house distance was 19 inches. A part of the slide where tissue was not present was photographed to give a background count: all 4 of the photographs were taken at 200 × magnification.

For counting the number of particles in the photographs, a mechanical scanner described by Kirsch, Cahn, Ray and Urban (1957) was connected to the SEAC computer at the National Bureau of Standards. This scanner reproduces within the computer a binary quantized replica of an area 44 mm. × 44 mm. in size on the original photograph. The scanned area is represented as a matrix of 176 × 176 squares each of $\frac{1}{4}$ mm. × $\frac{1}{4}$ mm. in dimension. For each $\frac{1}{4}$ mm. × $\frac{1}{4}$ mm. square, the total blackness is compared against an arbitrary fixed threshold value (manually set to be midway between that of a totally black and a totally white square). If the measured blackness is greater than this threshold, the square is treated as black, otherwise as white. For highly contrasted images, this quantizing procedure introduces an error whose magnitude is proportional to the total boundary between black and white areas, since for squares interior to a black or white region treating the squares as all black or all white is a correct representation of their real shade.

The counting program treats as separate objects only sets of black points with no connecting black points between them. The number of such disjoint sets of black points is the number of objects counted. Objects are thus defined entirely independent of any recognizable attributes of shape, size, or orientation.

Since each object will generally consist of many of the elementary $\frac{1}{4}$ mm. black squares, it is useful to count the total number of such squares. This was done in place of the more obvious determination of a size distribution because the objects as defined above need not constitute whole objects in any biological sense. For comparison of total area, the total size of all the objects should be compared against the size of the whole picture which is 176 × 176 = 30,976 of the $\frac{1}{4}$ mm. squares.

### RESULTS

The cellular details of the sections of rat stomach, duodenum and ileum were well preserved after having been incubated for 1 hour with tritiated 2-AF or 2,7-DAF. In most sections only mucosa was present; in some parts muscular layer was also seen. When comparing the density of the silver granules present in the sections, it was evident that the stomach sections contained more granules than the sections of the duodenum and that only few granules were present in the ileum regardless of whether the tissues were incubated with 2-AF or 2,7-DAF.

### EXPLANATION OF PLATE.

FIG. 1.—Fundus of the rat stomach incubated with 2-aminofluorene-$^3$H. This photograph which shows the cellular details was taken with phase contrast × 150 on ortho process contrast film and printed on illustrators azo. The white dots are the negative of the black dots shown in Fig. 2. The radioactivity is found on the cell membranes and between cells.

FIG. 2.—The same as in Fig. 1, except processed to show only the silver grains denoting radioactivity. Made with an 0·3 neutral density filter on Kodaliff film.

FIG. 3.—Fundus of the rat stomach incubated with 2,7-diaminofluorene-$^3$H. The other details are as given in Fig. 1. Cytoplasmic radioactivity.

FIG. 4.—Fundus of the rat stomach incubated with 2,7-diaminofluorene-$^3$H. The other details are as given in Fig. 2.

1

2

3

4

Ray, Rivers and Hoch-Ligeti.

The number of granules in the duedenum and ileum were about the same in the 2-AF and in 2,7-DAF treated sections. In the stomach, however, particularly in the fundal part, the number of granules in the 2,7-DAF treated sections greatly exceeded that in the 2-AF treated sections.

The microscopic appearance and the distribution of the silver granules in the fundal part of the gastric mucosa after incubation with 2-AF is shown in Fig. 1 and 2 ; that of 2,7-DAF treated gastric mucosa in Fig. 3 and 4. The automated particle count on Fig. 2 for an area 20 mm. $\times$ 20 mm. gave 226 as the number of dots. The total blackened area for 44 mm. $\times$ 44 mm. is 1363 $(\frac{1}{4})^2$ mm$^2$. On Fig. 4 for 20 mm. $\times$ 20 mm. the number of dots is 391. The total blackened area for 44 mm. $\times$ 44 mm. is 3101 $(\frac{1}{4})^2$ mm$^2$. The quantitative difference found confirms our previous *in vivo* results (Ray, Cromer, Aycock and Pitzer, 1961) with the acetyl derivatives. The microscopic analysis of these tissues shows that in the 2-AF treated stomach mucosa most of the particles, representing the effect of the tritiated compound, are located on the cell membranes or between the cells forming roughly a hexagonal pattern. In the 2,7-DAF treated stomach mucosa, particles are located also within the cytoplasm, appearing therefore more diffusely distributed. The nuclei appear to be free of these particles.

### DISCUSSION

The ability of a carcinogen to cause cancer in a particular organ undoubtedly depends on a number of factors, not all of which, perhaps, may be surely known. The inherent resistance or susceptability of a tissue to transformation into the neoplastic state is, of course, a prime factor but until the extrinsic factors are known this inherent property cannot be assessed. One of these factors is the ability of a carcinogen to reach the target tissue. Since the animal stomach does not develop adencarcinoma readily, it seemed to us that perhaps the carcinogens were not reaching the target in sufficient concentration.

From a study of gastric secretion by Ray and Pease (1957) it seemed likely that basic carcinogens would be successful in reaching the mucosa of the stomach This proved to be the case and both 2-acetylaminofluorene, AAF, and 2,7-diacetylaminofluorene, DAAF, were secreted by the stomach. The DAAF was more basic, was retained by the stomach to a greater degree, and was the only one to produce adenocarcinoma (Morris, Wagner, Ray, Stewart and Snell, 1962). Thus, a *quantitative* factor was a distinct possibility. In the present work we have shown that there seems, also, to be a *qualitative* difference in that 2-aminofluorene is found on the cell membranes or between the cells (Fig. 1 and 2). 2,7-Diaminofluorene, on the other hand, is located within the cytoplasm (Fig. 3 and 4). Since the processing of the slides should serve to remove all but the protein bound carcinogen it seems that we are justified in claiming as a first approximation that cytoplasmic protein binding is (in this case at least) a requisite factor in carcinogenesis.

From the experimental evidence here presented it seems probable that the conversion of 2,7-diaminofluorene to its metabolites and consequent elimination from the cell does not take place as readily as does 2-aminofluorene. The cytoplasmic protein binding has prevented the rapid elimination of the gastric carcinogen which in turn has led to the production of adenocarcinoma in the normally resistant rat stomach.

### SUMMARY

Stomach tissue of rats has been incubated with tritiated 2-aminofluorene or 2,7-diaminofluorene hydrochloride. The latter is a gastric carcinogen and the silver dots deposited in stomach sections as the result of $\beta$-radiation were considerably more numerous with that compound than with 2-aminofluorene. In sections incubated with 2-aminofluorene, the particles were presented generally on the cell membrane or between the cells while in the sections incubated with 2,7-diaminofluorene many particles were found also in the cytoplasm. The number and the distribution of the particles did not differ with the tritiated compounds in the sections of duodenum or ileum.

This investigation was supported by Public Health Service Research Grant No. Ca 07737–01 from the National Cancer Institute and the Charles McCamic Memorial Grant for Cancer Research from the American Cancer Society, No. P-2661. Our thanks are due to Mr. Russell Kirsch, Applied Mathematics Division, National Bureau of Standards, Department of Commerce, Washington D.C., for providing the automated analysis of particle counts and to Mr. Harry Busey, Chief Medical Photographer, Veterans Administration Center, Martinsburg, West Virginia, for help with photographic procedures.

### REFERENCES

ALFRED, L. J.—(1964) *Brit. J. Cancer*, **18**, 564.

BOYD, GEORGE A.—(1955) 'Autoradiography in biology and medicine', New York (Academic Press Inc.)

CON-RAD/JOFTES—(1960) 'Instruction manual for fluid emulsion radioautography system,' Cambridge 40, Mass. (Controls for Radiation, Inc.).

KIESCH, R. A., CAHN, L., RAY, L. C. AND URBAN, G. H.—(1957) *Proc. east. jt Computer Conf.* (Am. Fed. Inform. Proc. Soc.) pp. 221–229.

'Manual of Histologic and special staining technics'—(1960) Armed Forces Institute of Pathology, 2nd edition, New York. (The Blakiston Div., McGraw-Hill Book Co.) p. 134.

MORRIS, H. P., WAGNER, B. P., RAY, F. E., SNELL, K. C. AND STEWART, H. L.—(1961) 'Carcinogenicity of N,N'-2,7-fluorenylenebisacetamide'; National Institutes of Health Monograph No. 5. Washington (U.S. Govt. Printing Office).

MORRIS, H. P., WAGNER, B. P., RAY, F. E., STEWART, H. L. AND SNELL, K. C.—(1962) *J. nat. Cancer Inst.*, **29**, 977.

RAY, F. E., CROMER, M. A., AYCOCK, A. C. AND PITZER, N. H.—(1961) *Brit. J. Cancer*, **15**, 816.

RAY, F. E. AND JUNG, M. L.—(1951) *Brit. J. Cancer*, **5**, 358.

RAY, F. E. AND PEASE, P.—(1957) *Am. J. Physiol.*, **190**, 109.

STEWART, H. L., SNELL, K. C. AND HARE, W. V.—(1958) *J. nat. Cancer Inst.*, **21**, 999.